# *Monodelphis domestica* as a Fetal Intra-Cerebral Inoculation Model for Zika Virus Pathogenesis

**DOI:** 10.3390/pathogens12050733

**Published:** 2023-05-19

**Authors:** John Thomas, Juan Garcia, Matthew Terry, Susan Mahaney, Oscar Quintanilla, Dionn Carlo Silva, Marisol Morales, John L VandeBerg

**Affiliations:** 1 Center for Vector Borne Disease, The University of Texas Rio Grande Valley, Edinburg, TX 78539, USA; 2 Department of Biology, The University of Texas Rio Grande Valley, Edinburg, TX 78539, USA; garciajrj@livemail.uthscsa.edu (J.G.); matthew.terry@utrgv.edu (M.T.); oscarq0727@aol.com (O.Q.); dionncarlo.silva01@utrgv.edu (D.C.S.); moralesm20@livemail.uthscsa.edu (M.M.); 3 Department of Human Genetics, The University of Texas Rio Grande Valley, Brownsville, TX 78521, USA; susan.mahaney@utrgv.edu; 4 School of Medicine, The University of Texas Rio Grande Valley, Edinburg, TX 78539, USA; 5 South Texas Diabetes and Obesity Institute, The University of Texas Rio Grande Valley, Brownsville, TX 78521, USA

**Keywords:** Zika, fetal, Monodelphis, model, animal, intra-cerebral, immunocompetent

## Abstract

*Monodelphis domestica* (the laboratory opossum) is a marsupial native to South America. At birth, these animals are developmentally equivalent to human embryos at approximately 5 weeks of gestation, which, when coupled with other characteristics including the size of the animals, the development of a robust immune system during juvenile development, and the relative ease of experimental manipulation, have made *M. domestica* a valuable model in many areas of biomedical research. However, their suitability as models for infectious diseases, especially neurotropic viruses such as Zika virus (ZIKV), is currently unknown. Here, we describe the replicative effects of ZIKV using a fetal intra-cerebral model of inoculation. Using immunohistology and in situ hybridization, we found that opossum embryos and fetuses are susceptible to infection by ZIKV administered intra-cerebrally, that the infection persists, and that viral replication results in neural pathology and may occasionally result in global growth restriction. These results demonstrate the utility of *M. domestica* as a new animal model for investigating ZIKV infection in vivo and facilitate further inquiry into viral pathogenesis, particularly for those viruses that are neurotropic, that require a host with the ability to sustain sustained viremia, and/or that may require intra-cerebral inoculations of large numbers of embryos or fetuses.

## 1. Introduction

Zika virus (ZIKV) is a small, enveloped positive-sense RNA virus from the family *Flaviviridae*. Typically transmitted in a zoonotic cycle that alternates between a vertebrate host and an invertebrate vector, ZIKV gained notoriety following the 2015 outbreak in Brazil, which saw a dramatic increase in the number of neurological abnormalities in infants born to ZIKV-infected mothers [1]. Significant increases in Guillain-Barre syndrome and microcephaly during this outbreak were also observed when compared to previous years [2], perhaps fulfilling the theory posited by Hayes when he declared ZIKV to be neurovirulent [3].

Following the initial isolation of ZIKV from the upper canopy of the Ziika Forest in Uganda in 1947 [4], little research into the neuropathology of ZIKV had been carried out prior to the Brazilian epidemic. It is estimated that more than 400 babies were born with microcephaly and other brain abnormalities to ZIKV-infected pregnant women during this outbreak [5] and, subsequently, analysis of fetal tissue collected from ZIKV-infected infants supports a causal relationship between ZIKV and neurological abnormalities, as ZIKV has been detected in brain tissue of microcephalic fetuses as well as in amniotic fluid of pregnant women [6,7,8]. The dramatic increase in the incidence of microcephaly and other fetal abnormalities from the Brazilian outbreak has spurred the development of animal models of infection in order to study the effects of ZIKV replication in vivo, with a particular focus on the neurotropism of ZIKV. To date, the principal animal models for assessing ZIKV pathology have been nonhuman primates (NHPs) and transgenic mice; limited studies have also been conducted with chicken embryos [9]. 

The NHP model is the most relevant in terms of reproducing the pathology in vivo compared to what is known about ZIKV-induced pathologies in humans [10,11,12,13]. Macaques have been the NHP model used most frequently, and several studies have demonstrated the advantages of the NHP model by comparison with the mouse model, including similarities to human gestation, ease of studying placental transmission, and robust immune responses as expected in an immunocompetent animal model [10,14]. In a fetal macaque model, ZIKV elicited severe pathological effects on the central nervous system (CNS), including damage to the axonal and ependymal areas, gliosis, and hypoplasia of the cerebral white matter [13]. Other studies using NHPs have shown high viral loads in the sex organs and consistent viral shedding in the oral mucosa, further suggesting that NHPs may be uniquely suited to addressing many questions pertaining to ZIKV pathogenesis in vivo [10,14]. However, the cost associated with the use and maintenance of NHPs precludes large-scale experimentation. This limitation, together with the long duration of time required to investigate the effects of ZIKV infection during gestational development, infancy, adolescence, and into adulthood and old age in NHPs, suggests that additional animal models are required. 

Studies conducted with immune-deficient murine models have demonstrated the ability of ZIKV to replicate in neuronal and ocular tissue [15,16], to delay development and whole-body growth [17], to reduce cortical thickness and cell numbers [17], and to elicit apoptosis in ZIKV-infected neurons [18]. Many of the immune-deficient murine models are based on the abatement of type I interferon responses (A129 mice) or types I and II interferon (AG129 mice) [19]. These animals are highly susceptible to ZIKV infection, maintain a high viral load in the CNS, and demonstrate the ability of ZIKV to infect cells associated with the testes, an observation that is consistent with the findings of sexual transmission of ZIKV from males to males and to females in humans [20,21]. The transgenic murine models have generated useful data regarding ZIKV pathogenesis; however, because these animals are deficient in cell-mediated immune responses that are often the most effective defense against intracellular pathogens [22], the data from transgenic murine models may not be fully representative of the pathology observed in humans. 

Other studies have used subcutaneous ZIKV infection in immunocompetent 1-day-old C57BL/6 pups (immunocompetent to the limited extent that 1-day-old mouse pups have begun to develop their immune system), which resulted in the development of major brain abnormalities including neuronal cell death, gliosis, and axonal rarefaction [23], all of which are representative of ZIKV replication in human brain tissue [2,24,25]. From a developmental perspective, however, a 1-day-old mouse pup is approximately equivalent to a human fetus at 19 weeks of gestation [26] and, as such, is not suitable for modeling the effects of ZIKV infection on human embryonic and earlier fetal stages. Moreover, C57BL/6 pups inoculated at 3 or 10 days of age did not develop any signs of disease, so the neonatal mouse model is limited to a single stage of fetal development.

In an effort to model ZIKV infection at those stages of human development, Shao et al. [27] performed intra-cerebral inoculations at 14.5 days of gestation (e14.5) with ZIKV and allowed them to develop. An e14.5 mouse embryo is developmentally equivalent to a human embryo at 7–8 weeks post-conception [26]. Massive neuronal death occurred in the inoculated embryos, and although some of them survived to birth, the oldest animal reported was 3 days old, suggesting that the infection is lethal within days of birth. Due to the time and effort required for inoculating mouse embryos, this model is not practical for high-throughput experiments that are required for modeling the various potential outcomes of human embryo infection with ZIKV. Moreover, since the infection is lethal in this model, it is not possible to use it to investigate the long-term sequelae of ZIKV infection at the embryonic stage.

Because all of the existing animal models of ZIKV-induced pathogenesis have significant limitations, we explored the potential of a marsupial model to circumvent those limitations. The gray short-tailed opossum, *Monodelphis domestica*, is native to Brazil and surrounding countries. The laboratory genetic stocks and inbred strains of this species are collectively referred to as the laboratory opossum [28]. Laboratory opossums are widely used as models in many fields of biomedical research [28,29], and they possess some characteristics that render this model suitable, and in some respects, unique, for studying the pathogenesis of ZIKV in vivo. First, the animals are fairly small (80–140 g as adults), but several times the size of a mouse, facilitating some experimental procedures by comparison with mice, such as serial collections of substantial quantities of blood. Second, they are highly fecund and easy to manipulate, and they can be produced and maintained cost-effectively. Third, at birth, *M. domestica* are developmentally equivalent to a human embryo at approximately 5 weeks of gestation [26], and they complete embryonic and most of fetal development while attached to the mother’s nipples over a 2-week period [29]. Fourth, female *M. domestica* do not have pouches, so the pups can easily be experimentally manipulated while they are attached to the nipples, and they have a high rate of survival post-manipulation. Fifth, while the immune system is undeveloped at birth, *M. domestica* develops a fully intact immune system as they progress beyond the fetal stage. Last, as is also true for the immune-deficient murine models but not for the in utero murine model or the NHP model, large numbers of *M. domestica* can be used economically, enabling robust statistical analysis for between-group comparisons as well as robust assessment of within-group variations in the outcome of ZIKV infection. 

We emphasize the importance of being able to assess within-group variation in large numbers of animals inoculated with ZIKV for the purpose of modeling the major variations in the pathological outcome of human infection with ZIKV. For example, (1) growth retardation and microcephaly are uncommon outcomes of ZIKV infection of human embryos and fetuses [24]; (2) eye pathologies occur in only a minority of children who were infected in utero and in only a small proportion of children and adults who become infected with ZIKV [6,30]; and (3) Guillain-Barre syndrome is caused by ZIKV infection in only a minority of people [31]. 

The purpose of this study was to assess the utility of *M. domestica* as an intra-cerebral model for ZIKV neuropathogenesis by determining if ZIKV can replicate and persist in the brains of young pups and, if so, to determine the nature and extent of the neuropathological consequences by comparison with those observed in humans and other animal models. 

We point out that the experiments reported here are not intended to model the complex biological processes that lead to infection of the brains of human embryos and fetuses with ZIKV, typically beginning with the bite of a mosquito, replication in the mother, trans-placental transfer to the embryo or fetus, replication in the embryo or fetus, followed by entry into the brain and replication in the brain. Rather, our model obviates many of the variables and mechanistic complexities that exist between the time of initial infection in the mother and the entry of the virus into the brain of the embryo or fetus. Via the use of this unique model, we can conduct high-throughput experiments to investigate the short-term and long-term pathological effects of variation in the number of PFU that enter the brain, the exact developmental time point at which they enter the brain, and the genetic make-up of different ZIKV strains in the absence of the many confounding variables that exist in models of trans-placental infection of embryos and fetuses.

## 2. Materials and Methods

### 2.1. Animals 

The laboratory opossums used in this study were produced in the breeding colony maintained at the University of Texas Rio Grande Valley and maintained under standard conditions [28]. Briefly, the animals were maintained in individually ventilated cages (Animal Care Systems): Optirat cages for mated pairs, mothers with litters, and groups of weaned littermates up to 4 months of age; and Optimice cages for individually housed animals beyond four months of age. The animals were fed Purina LabDiet^®^ Short-Tailed Opossum #2, 5ATD, chow ad libitum, and water purified by reverse osmosis. Paired animals and group-housed littermates were provided with nest boxes, and all animals were provided with shredded paper towels for nest building. Euthanasia was performed with carbon dioxide administered by the gradual fill method.

### 2.2. Cells and Viruses

ZIKV isolate PRVABC59 (a gift from Dr. Kenneth Plante at the WRCEVA repository at UTMB) was used for the inoculations. Vero cells (CCL-81; American Type Culture Collection, Manassas, VA, USA) were used for virus titration, and C6/36 cells (CRL-1660; American Type Culture Collection, Manassas, VA, USA) derived from *Aedes albopictus* were used to amplify lyophilized virus for scale-up. The virus generated from the initial reconstituted lyophilized stock was passaged once in C6/36 cells, and the resulting supernatant was clarified and purified over a sucrose cushion. Virus supernatants were quantified in duplicate by plaque assay, as described previously [32]. Aliquots were stored at −80 °C for further use. 

### 2.3. Susceptibility of M. domestica Pups to ZIKV Infection

In the first experiment, *M. domestica* pups (n = 6) were inoculated with 5000 PFU of ZIKV PRVABC59 intra-cerebrally. Two litters at 1 and 2 days of age, respectively, were used, hereafter referred to as Group 1 and Group 2. Each group contained three animals. At 20 days post-inoculation, the animals were euthanized, and whole brains were collected, weighed, and homogenized for virus titration in 1.0 mL of DMEM containing 2% FBS (Gibco, Waltham, MA, USA). Next, the supernatants were collected by centrifugation at 8000 rpm for 15 min at 4 °C and frozen at −80 °C. Supernatants from the homogenized brain samples were quantified in duplicate by plaque assay, as described previously [32].

### 2.4. Developmental Effects of Intracerebral Inoculation 

Following the initial confirmation that *M. domestica* pups could be infected with ZIKV via the intra-cerebral route, in the second experiment, we examined the effects of ZIKV infection on postnatal development in the laboratory opossum model. *M. domestica* pups (n = 16) ranging in age from 4–20 days (equivalent in human development to 8–20 weeks post conception) were inoculated intra-cerebrally with 5000 PFU of ZIKV as described above. Control animals (n = 10), ranging in age from 2–9 days, were inoculated with PBS. Seventy-four days after the inoculations, the animals were euthanized, weighed, and measured; and brain tissue was collected for analysis by immunohistochemistry and in situ hybridization. 

### 2.5. Tissue Fixation and Sectioning

Dissected tissue was fixed in sterile PBS (Gibco, Waltham, MA, USA) + 4% formaldehyde solution and stored at room temperature. Fixative was then cleared from tissue by performing three quick washes in sterile PBS, followed by three 10-min washes in sterile PBS. Next, the tissue was washed 1X for 5 min in a 25% methanol:PBS solution; washed 1X for 5 min in 50% methanol:PBS; and finally washed 3X for 5 min in 100% methanol. Tissue was stored at −20 °C until needed. The tissue was rehydrated by washing 1X for 5 min in 50% sterile methanol:PBS; washing 1X for 5 min in 75% methanol:PBS; and then washing 3X for 5 min in sterile PBS. Tissue was incubated for 30 min in 33% OCT mounting media: sterile PBS; 30 min in 66% OCT: sterile PBS; and 1–4 h in 100% OCT. Tissue was mounted in OCT and cooled to −20 °C for sectioning by a cryostat (Leica Biosystems, Wetzlar, Germany). Sections of 5–10 µm were mounted onto Frost + microscope slides and stored at −20 °C.

### 2.6. Antibody Staining

Mounted sections of tissue were incubated in PBTB (sterile PBS + 0.01% Tween20 + 0.2% BSA) for 1 h, followed by incubation in a 1:500 dilution of the primary antibody (Arigo Biolaboratories, Hsinchu City, Taiwan) for either 1 h at room temperature or overnight at 4 °C. Primary antibodies were removed by washing 3X quickly, then 3X for 10 min each in PBTB. Tissue was then incubated in a 1:200 dilution of AlexaFluor (546 or 647—Thermo Fisher Scientific, Waltham, MA, USA) conjugated secondary antibody in PBTB for 1 h. Secondary antibody was removed in the same manner as primary antibody, except that DAPI (Thermo Fisher Scientific, Waltham, MA, USA) and AlexaFluor 488 (Thermo Fisher Scientific, Waltham, MA, USA) conjugated phalloidin were included in the first 10-min wash. Tissue was imaged using an Olympus FV10i confocal microscope (Olympus Microscopes, Tokyo, Japan).

### 2.7. RNA Probe Preparation

Zika virus strain ZikaSPH2015′s complete genome (Accession #KU321639; Version# KU321639.1) was used as the parental genetic base. Probe sequences were derived from alignments with multiple strains of ZIKV using Clustal (www.Clustal.org; accessed 1 March 2018). Target genes were amplified using standard PCR and then cloned into the PCRII^®^ Expression vector (Invitrogen, Waltham, MA, USA) as per the manufacturer’s instructions. Cloned products were verified via DNA sequencing and then linearized by a second PCR using M13F and R primers. After standard PCR cleanup, the linearized gene was quantified and then normalized to 100 ng/µL. Digoxigenin (DIG)-tagged probes were made using the SP6 and T7 promoters to make either sense or antisense probes in separate reactions using the following mix: 200 ng linearized cloned PCR product, 2 µL 10× transcription buffer, 1 µL of 0.1M DTT (0.02 M DTT for SP6 reaction), 2 µL of DIG-labeled ribonucleotides, 1 µL of RNase inhibitor, and 1 µL of either SP6 or T7 polymerase (New England Biolabs, USA). SP6 reactions were incubated at 40 °C for 2 h, and T7 reactions were incubated at 37 °C for 1 h. Successful probe synthesis was confirmed via standard gel electrophoresis, and probes were cleaned using ethanol precipitation and re-suspended in 50 µL of DEPC H_2_O, quantified via spectrophotometry, and stored at −80 °C. Zika NS5 Protein Forward, Zika NS5 Protein Reverse, and NS5 Probe Sequences are shown in Supplemental Figure S1. 

### 2.8. In Situ Hybridization

Mounted sections of tissue were incubated in RNase-free PBST (sterile PBS + 0.01% Tween20) for 5 min, 50% PBST: hybridization buffer (50% formamide, 5X SSC, 100 µg/mL salmon sperm, 0.1% Tween20, 100 µg/mL heparin) for five min, then in hybridization buffer for 5 min. A new hybridization buffer was placed on the sections, and pre-hybridization was performed in a small, airtight container at 56 °C for 2 h. The working probe solution was prepared by adding ~200 ng of probe to 100 µL of hybridization buffer and then incubating at 90 °C for 5 min, after which the incubation tubes were placed on ice. The working probe solution was then applied to tissue sections and incubated in an airtight container at 56 °C for 16–24 h. Probes were washed from the sections using a variety of wash times and numbers, with all washes using hybridization buffer warmed to 56 °C and all washes conducted at 56 °C. The following protocol was used to minimize non-specific signals: eight washes of 15 min each, followed by four washes of 30 min each. The slides were then cooled to room temperature and washed 1X for 5 min in 50% PBTB: hybridization buffer, 3X for 5 min each in PBTB, and 1X for 1 h in PBTB (to block non-specific protein binding). Slides were then incubated either for 1 h at room temperature or overnight at 4 °C in a 1:100 dilution of an HRP-conjugated anti-DIG antibody and PBTB. This antibody was removed by three quick washes and then three 5-min washes in PBTB. Slides were washed in 1X tyramide buffer for 5 min. Fluorescent labeling was performed using the AlexaFluor Superboost^®^ tyramide signal amplification kit (Thermo Fisher Scientific, Waltham, MA, USA), following the manufacturer’s instructions, and using either the 546 or 647 markers. After the tyramide reaction was stopped, excess reagent was removed by washing 3X quickly, then 3X for 10 min each in PBTB. DAPI and AlexaFluor 488 conjugated phalloidin were included in the first long wash to label nuclei and cytoskeletal elements, respectively. The tissue was imaged using an Olympus FV10i confocal microscope.

### 2.9. Pathology and NS1 Scoring of Tissues

Brain slices from all animals were prepared, stained, and visualized for detection of NS1 as described above. Tissues were then scored based upon the pathology of the tissue as well as the expression of NS1. Brain pathology was scored subjectively on a scale of 0–3: 0, normal; 1, mild pathology; 2, moderate pathology; 3, extreme pathology. Brain NS1 levels (extent of fluorescent signal) were scored similarly, using the nuclei visible within the field of view at 60×: 0, none; 1, minimal; 2, moderate; 3, extreme. Images from the PBS control animals were used as an example of normal, uninfected tissue and established a baseline representation score of 0 (normal morphology; no NS1 signal).

### 2.10. Analysis of Apoptosis

Brain slices from all animals were prepared, stained, and visualized for the detection of apoptosis as described above. A TUNEL assay kit (Abcam, Waltham, MA, USA) was used to detect DNA fragmentation using fluorescent microscopy. Briefly, the sections were rehydrated through a descending ethanol series: 2 × 3 min in 95% EtOH; 1 × 3 min in 90% EtOH; 1 × 3 min in 80% EtOH; and 1 × 3 min in 70% EtOH; followed by a wash step of 3 min using ddH20. Next, the tissues were incubated with proteinase K for 5 min at room temperature and then refixed with 5% formaldehyde and washed in ddH20. Slices were then incubated in the DNA labeling solution for 60 min at 37 °C, and then another ddH20 wash was performed. Lastly, the slices were incubated in the anti-BrdU antibody solution for 30 min at room temperature, washed 3X, and 7-AAD was added to label nonviable cells and incubated for 30 min at room temperature. Cells were visualized using fluorescence microscopy with an Olympus FV10i confocal microscope.

## 3. Results

### 3.1. Susceptibility of Monodelphis Domestica to ZIKV Infection

Viral replication was detected in two of the three animals from Group 1 (1 day old at the time of inoculation) and one of the three animals from Group 2 (2 days old at the time of inoculation). The average titer was 1.8 × 10^4^ PFU/g of brain tissue from the two 1-day-old pups in which the virus was detected, while the titer from the single 2-day-old pup that was infected was 4.3 × 10^4^ PFU/g of brain tissue (Table 1).

### 3.2. Developmental Effects of Intra-Cerebral Inoculation 

**Figure 1:**
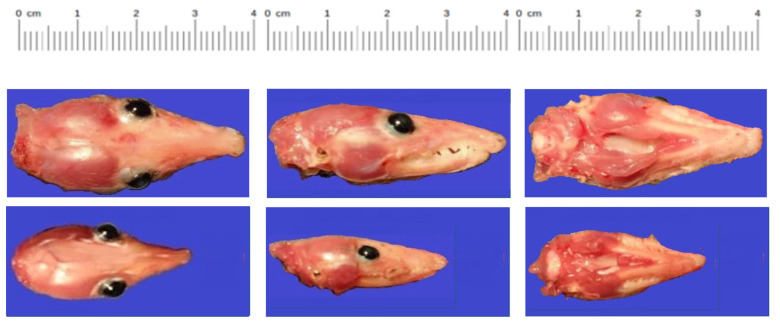
Heads of normal (**top**) and growth-restricted (**bottom**) *M. domestica* littermates at 80 days of age. Six-day-old *M. domestica* pups from a single litter were inoculated with 5000 PFU of ZIKV PRVABC59 intracerebrally. At 74 days post-infection (80 days of age), the animals were euthanized, and photographs and measurements (Table 2) of the heads were taken.

One animal (O9355) among five littermates that were inoculated at 6 days of age and euthanized at 80 days of age had much lower values for body weight, body length, and head length and width compared to those of its littermates (Table 2; Figure 1). None of the other 10 ZIKV-inoculated animals exhibited growth abnormalities. During 40 years of producing nearly 150,000 laboratory opossums that were not inoculated with ZIKV, we have not observed another animal with such severe growth restriction. Furthermore, none of the 10 PBS-inoculated animals exhibited growth restriction.

### 3.3. The Presence of Viral Protein and RNA in Brains Infected with ZIKV

**Figure 2:**
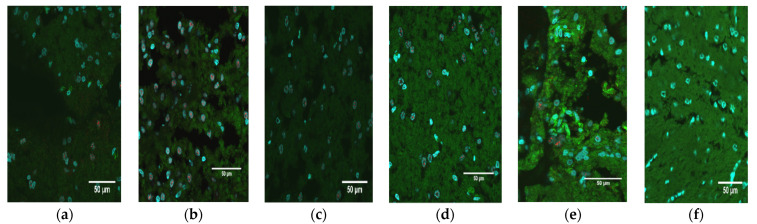
Immunohistochemical detection of ZIKV. (**a**) Immunofluorescence staining of a transverse section of the cerebellum from an infected growth-restricted pup (O9355) at 60× with an anti-ZIKV NS1 monoclonal antibody (red). The cytoskeleton is stained green; the nuclei are blue. (**b**–**e**) Cerebellum section from infected littermates (O9356–O9359). (**f**) Cerebellum section from a mock-infected animal (O9341) at 60×.

**Figure 3:**
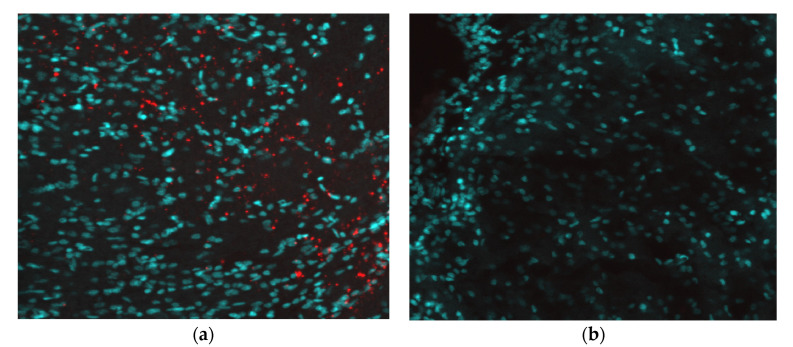
In-situ hybridization detection of ZIKV RNA. (**a**) Immunofluorescence staining of a transverse section of the cerebellum from an infected growth-restricted pup (O9355) at 60× showing the presence of ZIKV NS5 mRNA (red); the nuclei are blue. (**b**) Cerebellum slice from a mock-infected animal (O9339) at 60×.

The brains of the pups were fixed, sectioned, and stained for the presence of ZIKV NS1 protein. Immunofluorescence microscopy showed that, in brains collected from all 16 ZIKV-inoculated animals, anti-ZIKV monoclonal antibodies directed against NS1 bound specifically to neuronal cells, indicating that the brains were infected with ZIKV (Figure 2a–e). The number of cells visibly expressing NS1 was evaluated and scored based upon the number of nuclei displaying a characteristic punctate staining pattern that we observed in all infected neural tissue samples (Table 2; Supplemental Figure S2). All infected animals showed the presence of NS1 within the brain sections, and the amount of signal appeared to correlate to the pathology of the tissue. Animals that showed the most severe pathology also had the highest number of NS1-fluorescing nuclei, while the samples with more moderate and low pathology scores had low to moderate levels of NS1 expression (Table 2). Spearman’s correlation coefficient between these semi-quantitative measures of brain pathology and the NS1 signal is 0.59 (*p* = 0.008) for a 1-tailed test of the hypothesis that the correlation would be positive. Brain tissue sections from each of the 10 animals inoculated with PBS exhibited no evidence of ZIKV (Figure 2f). To further confirm the presence of ZIKV replication, an in-situ hybridization assay was conducted on brain sections of O9355 to detect ZIKV vRNA using NS5 as a target gene. The results showed a strong signal for ZIKV NS5 RNA in the cerebellum (Figure 3). 

### 3.4. Pathological Consequences of ZIKV Infection in the Brain

**Figure 4:**
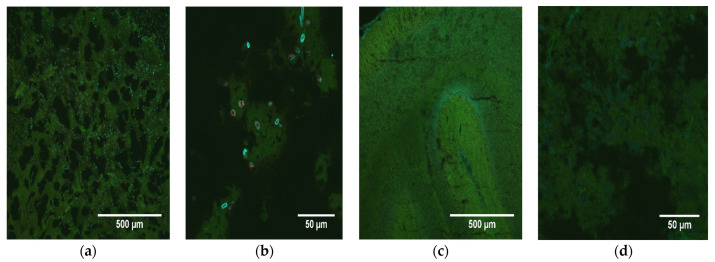
Spongiform morphology induced by ZIKV infection. (**a**) Immunofluorescence staining of a transverse section of the cerebellum from an infected pup (O9251) at 10× showing a spongiform-like pathology in the presence of ZIKV NS1 protein (red). The cytoskeleton is stained green; the nuclei are blue. (**b**) Cerebellum from the same animal at 60×. (**c**) Cerebellum from a mock-infected animal (O9339) at 10×. (**d**) Cerebellum from the same mock-infected animal (O9339) at 60×.

**Figure 5:**
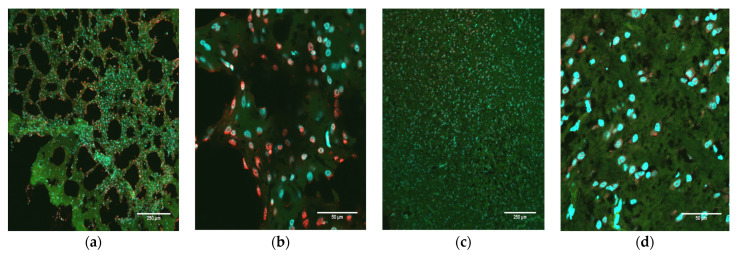
Apoptosis as a consequence of ZIKV infection. (**a**) Immunofluorescence staining of a transverse section of the cerebellum from an infected pup (O9336) at 10× showing a spongiform-like pathology in the presence of apoptosis (red). The cytoskeleton is stained green; the nuclei are blue. (**b**) Cerebellum from the same animal at 60×. (**c**) Cerebellum from a mock-infected animal (O9396) at 10×. (**d**) Cerebellum from the same mock-infected animal (O9396) at 60×.

In addition to demonstrating the presence of ZIKV RNA and protein in the brains of infected pups, images of the fixed neural tissues collected from infected opossum pups were also evaluated based upon the observable cell morphology and scored for severity of disease (Table 2). Most of the brain slices showed either a mild or moderate pathology; however, three samples displayed a discontiguous, spongiform-like pathology with large gaps between cells (i.e., a dramatic reduction in density of cells), along with large clumps of DAPI-stained (blue) DNA, which apparently had been released from cells as they died and which had aggregated into large extra-cellular clumps (Figure 4a). Further examination of brains with this spongiform morphology showed the presence of high levels of ZIKV NS1 protein (Figure 4b). The cerebellum slices from the PBS-inoculated control animals had a uniform, contiguous appearance with little to no gaps between cells, no apparent destruction or cell death, and no extracellular DNA (Figure 4c,d). When the tissues were analyzed for DNA fragmentation, a hallmark of apoptosis, clear differences were seen between control animals and those infected with ZIKV (Figure 5). In animals intracerebrally infected with ZIKV (Figure 5a,b), cerebellum slices showed strong signals suggesting apoptosis. However, control animals inoculated with PBS (Figure 5c,d) showed little apoptosis, which was comparable to that observed in a baseline analysis of uninfected cerebellum slices (data not shown), suggesting that cerebellar ZIKV infection induced an apoptotic response in neuronal cells that was markedly higher than the apoptotic response in uninfected animals. 

## 4. Discussion 

Two critical questions that pertain to the development of a new animal model of infection are: (1) is the target host organism susceptible to infection and replication of the pathogen; and (2) does the pathology presented in the animal model accurately reproduce at least some of the clinical findings seen in cases of human infection? As mentioned above, flaviviruses such as dengue virus (DENV) and Zika virus (ZIKV) grow poorly, or not at all, in non-primate animals with intact immune systems [9]. Indeed, this lack of susceptibility to viral infection has led to the development and use of immunocompromised transgenic mice and chicken embryos as potential models for ZIKV infection [9]. While these models have demonstrated some utility within the context of understanding ZIKV biology, abatement of the primary immune responses directed against viruses for the purpose of establishing infection may hinder the interpretation of results within the context of relevance to human subjects. Normal, wild-type immunocompetent 1-day-old mice (to the limited extent that mice have a competent immune system at that early age) have been used to model aspects of ZIKV replication and pathology [16]; however, 1-day-old mice correlate with a human fetus at 19 weeks of gestation (20 weeks is mid-gestation) [4]. In contrast, a newborn *M. domestica* pup developmentally correlates to a human embryo at five weeks of gestation, thus allowing for ZIKV infection in newborn opossum pups to better replicate the pathology in the developing human embryo during the time when cellular differentiation in critical areas such as the brain is at an early stage. Therefore, the laboratory opossum model, in which ZIKV infection at the embryonic or early fetal stage can persist long term and which can be used experimentally in large numbers, is capable of contributing to our understanding of ZIKV-induced pathologies similar to those that are initiated in humans at early developmental stages.

Due to the potential severe consequences of ZIKV replication in the human brain and its causal association with neurological diseases such as microcephaly, encephalitis, and Guillain-Barre syndrome [24], the ability of *M. domestica* pups to support viral replication in neuronal tissue is an important first step in the validation of the ZIKV laboratory opossum model. The intra-cerebral route was chosen in preference to other routes of infection to directly assess the neurocytopathic effects of ZIKV replication in the developing brain in the absence of other variables that might affect viral burden, such as immune-mediated clearance of a bloodborne pathogen, which could limit the pathological response in some animals. Moreover, for this first investigation of ZIKV infection in *M. domestica*, we chose a route that would be expected to most easily facilitate infection in the absence of intrinsic factors that can work to limit access to the brain by viruses, such as the Blood Brain Barrier (BBB) [33,34]. Viral amplification of ZIKV and its long-term persistence following a single intracerebral inoculation of 4- to 20-day-old animals demonstrated that: (1) brain cells of this species are permissive to ZIKV replication; and (2) this replication ultimately results in cell death and tissue degradation. The ability of fetal *M. domestica* to support viral infection via the intra-cerebral route is not surprising, as fetal mouse brains also support ZIKV infection [15,17]. However, the long-term survival and continued replication of ZIKV in the brains of *M. domestica* inoculated as embryos or fetuses was a profoundly different outcome from that which occurs with mice. Analysis of the fixed neural tissue showed the presence of ZIKV NS1 protein throughout the tissue as well as massive cellular death in the brain compared to age-matched sham-inoculated control animals. The presence of NS1 and its distribution across the cerebellum show that ZIKV replication was persistent for 74 days beyond the inoculation of the virus and suggest that neuronal cells in varied states of differentiation were exposed to ZIKV. This result could explain the global growth restriction we observed in one animal and would be consistent with the selective neuronal vulnerability to ZIKV observed in humans [7]. The lack of correlation between the growth-restricted animal (O9355) and the pathology and NS1 scores (1 and 2, respectively) was somewhat surprising, as one would expect such a dramatic reduction in overall body size to be reflected in the brain tissue pathology and NS1 expression. One possible reason for this finding could be the roles that host genes play in ZIKV susceptibility and infection. Manet et al. showed that mice with broad genetic diversity displayed a variety of phenotypes across the spectrum of responses to ZIKV infection, from total resistance to severe symptoms and death, with large variations in replication kinetics, viral load, and brain tissue pathology [35]. The *M. domestica* used in this investigation were from several genetically diverse stocks, and O9355 was from a random-bred stock, so the animals were highly variable genetically. Another possible reason is that the inoculum was not delivered to any specific region of the brain, and, in addition, the virus dispersion within the brain may have led to significant differences in its concentrations among locations in the brain within and between animals. It could be that the pathology and NS1 scores of O9355 would have been higher in other brain locations that had a greater influence on growth than in the location that was sampled.

The NS1 protein of ZIKV is a homodimer that, based upon predicted and known NS1 genetic sequences for other flaviviruses, interacts with a variety of host immune factors [36,37] and is the major antigenic marker of flavivirus infection [37]. The intracellular form of NS1 is central to viral replication, whereas secreted and membrane-bound NS1 have been implicated in the excitation of the immune response [37]. The detection of high levels of ZIKV NS5 RNA 74 days post-infection in the one animal (O9355) examined by in situ hybridization confirms that the presence of NS1 detected by immunohistochemistry in the brains of all ZIKV-inoculated pups reflects persistent, active infections in the cerebellum at the time of euthanasia.

A critical finding of our study was the correlation between pathology and the level of NS1 signal in the brains of ZIKV-inoculated pups. We consider the correlation of 0.59 (*p* = 0.008) to be exceptionally high, given that these continuously distributed phenotypes were each subdivided into discrete categories (four for pathology, ranging from 0 to 3; and three for NS1, ranging from 1 to 3, since no ZIKV-inoculated pups scored 0 for the presence of NS1) and given the small sample size. The only three animals that had brains with a spongiform-like pathology (score of 3) all also had the highest score (i.e., 3) for NS1. The only two ZIKV-inoculated pups that had no observable brain pathology (score of 0) had the lowest score for NS1 (i.e., (1) for animals that were inoculated with ZIKV). These results establish that (1) some animals in which ZIKV has been present in their brains since the embryonic or fetal stage exhibit no obvious brain pathology; (2) there is variation among littermates in the extent of NS1 detected in the brains and consequent extent of pathology; and (3) pups as young as 4 days of age and pups as old as 20 days of age at the time of inoculation can develop severe (spongiform-like) brain pathology. Those ages are developmentally equivalent to humans at 8 weeks post conception to 20 weeks post conception (i.e., mid gestation) [4].

Another critical finding was the reduction in overall body size of one infected animal compared to its ZIKV-infected littermates or mock-infected control animals. While the sample size was small, the physical measurement data suggest that infection of *M. domestica* with ZIKV at the embryonic stage of development can occasionally result in severe growth restriction. Infection of immunocompetent mouse embryos can also result in growth restriction, and it has been suggested that infection of embryonic mouse brains by ZIKV causes an immune response that disrupts neurovascular development [27]. While initial reports from the WHO and CDC originally highlighted microcephaly as the major concern with vertical transmission of ZIKV infection in pregnancy, more recent studies refer to Congenital Zika Syndrome (CZS), of which microcephaly is one severe manifestation of infection [38]. ZIKV infection of a single pregnant pigtail macaque resulted in several sequelae in the fetus reminiscent of CZS in humans, including restricted fetal brain growth and the presence of viral RNA in the brain [12]. Additionally, infection of pregnant rhesus macaques similarly demonstrated evidence of disrupted fetal growth, prolonged maternal viremia, and inflammation at the maternal-fetal interface, including mild decidual perivascular inflammation (not unusual in human decidua) and placental acute chorioamnionitis [13,14]. Therefore, evaluation of the opossum model using the expanded criteria of CZS (as has been suggested by others to include, but not be limited to, microcephaly) may allow for a more comprehensive understanding of the neurotropism of ZIKV in the fetal brain. In the opossum model, we observed several manifestations of CZS, including: (1) an overall reduction in total body size and weight of one animal; (2) the presence of ZIKV NS1 protein as well as NS5 vRNA in the brains of infected pups; and (3) reductions in total numbers of brain cells. As discussed above, we also observed a spongiform-like pathology in three infected animals and higher levels of apoptosis among infected animals than in control animals. Together, these data suggest that ZIKV infection in the cerebellum was probably the cause of neural tissue degradation and apoptosis. Studies performed to assess the role of apoptosis in neuronal cells have suggested that ZIKV infection utilizes multiple mechanisms, including increased cell death, to elicit a reduction in neural progenitor cell volume and neuronal layers, which results in a condition that resembles microcephaly [27,31].

While it is unknown what the long-term sequelae of CZS would be in the opossum model, studies are underway to evaluate the long-term impact of ZIKV infection on growth, development, mental and physical capabilities, and behavior. Indeed, it has been recently shown that postnatal infection with ZIKV resulted in sustained structural and functional alterations in an infant macaque model [11]. This result suggests that ZIKV infection can have deleterious developmental implications that go far beyond the ‘classical’ definition of ZIKV neuropathology in relation to the size and structure of the brain. Indeed, we have demonstrated that subcutaneous, intramuscular, or intraperitoneal inoculation of ZIKV into juvenile laboratory opossums with intact immune systems can result in chronic infection, viral dissemination to many organs, including the brain and reproductive organs, and anatomic and histological abnormalities (unpublished data).

And, as shown recently, some of the symptoms described in the transgenic murine fetal models do not result in the development of microcephaly, suggesting that there may be other factors that influence neurological outcomes in transgenic murine models. For example, studies using pregnant C57BL/6 (Ifnar1-/-) dams infected with ZIKV showed fetal brain damage in the pups; however, no progression to microcephaly was observed [23].

The introduction of ZIKV to the Americas has been followed by a steady spread of the virus, tied to the range of the arthropod vector, which has also increased in recent years [39]. While ZIKV infection rates are certain to rise and fall cyclically, dependent at least in part on weather patterns (particularly rainfall patterns and consequent mosquito density), it is expected that the overall incidence of human infection will increase as more people are exposed to ZIKV via the bites of infected mosquitoes. Control methods are currently focused on the reduction or elimination of relevant vector populations, including the deployment of genetically modified mosquitoes in order to reduce vector populations [40]. In addition, the development and testing of several putative vaccine candidates have also begun [41]. While these techniques probably represent the best-case approach for dealing with ZIKV, the release of genetically modified organisms is a topic that requires intense study and oversight by the FDA before it is approved. Furthermore, efforts to develop and license a ZIKV vaccine will require at least several more years before such a product can become commercially available. As such, the development and characterization of the major aspects of ZIKV biology, including the neurovirulence and interactions between the immune system and ZIKV, will be required in order to fully support vaccine and drug design. While no animal model may offer a complete solution to understanding ZIKV biology, the *M. domestica* model offers unique opportunities to study the effects of CZS in a system that better represents human immunology and pre/post-natal interactions while allowing for statistically meaningful studies with large numbers of immunocompetent animals.

In summary, using the intra-cerebral route of inoculation, we infected *M. domestica* pups at embryonic and early fetal stages of development, and 74 days later, well beyond the age of weaning (56 days), we observed ZIKV replication and consequent pathogenesis in neuronal tissue. One infected animal exhibited significant retardation in body and head growth. Its four infected littermates appeared to be anatomically normal, as did the other 11 animals that had been inoculated with ZIKV, as well as all 10 animals inoculated with PBS.

These data suggest that laboratory opossums can be an important new model for studying the effects of ZIKV replication in vivo and perhaps also for testing drug therapies, vaccines, and other strategies for preventing pathologies caused by ZIKV infection. Moreover, it is possible that ZIKV persists long-term in the brains (an immunologically privileged site) of some humans after in utero infection, as it does in opossums, without causing any anatomical developmental abnormalities. If they do, some of them might develop brain pathologies, as some opossums do, and some of them might not develop brain pathologies.

The opossum that exhibited growth retardation was timid and tended to hide from its littermates, but the other infected opossums did not exhibit any noticeable gross abnormalities in behavior. However, it seems likely that at least those opossums with severe brain pathologies would exhibit abnormalities in behavior, social interactions, cognitive abilities, and memory if they were allowed to develop and were rigorously assessed. Recently, it has been demonstrated that some children who were exposed in utero to ZIKV and were not born with CZS exhibit delayed neurodevelopment and neurosensory alterations by the second year of life [42], and that some may have altered “executive function, mood, and adaptive mobility” by ages 3–5 years [43]. As a practical, non-genetically modified animal model that has a relatively short lifespan and can be utilized in large numbers, the opossum may prove to be critical for research on the long-term effects of in utero ZIKV infection on childhood development and into adulthood. It might also enable the development of strategies to mitigate those adverse outcomes.

## 5. Limitations of the Study

Our results suggest that intra-cerebral infection of *M. domestica* with ZIKV results in viral replication and virally mediated death of infected neural cells and can, on occasion, elicit growth retardation. There are, however, important factors to consider when interpreting these results. First, the reduction in growth was only observed in a single animal, so the model (at this stage of its development) yields only a small percentage of animals with an outcome that mimics CZS (as is consistent with the outcome of human in utero infections with ZIKV). Another important caveat is that, while we detected the presence of ZIKV among all infected animals, as confirmed by immunohistochemistry and in-situ hybridization, there were noticeable differences between animals that were within the same litter with regard to neuropathogenesis: some animals displayed significant pathology, while others presented tissues that were largely undamaged even though the dose and strain of ZIKV used were identical. Due to these factors, we relied on averaging observations and data from multiple animals to draw our conclusions. Finally, administration of ZIKV intra-cerebrally was technically challenging, as the newborn pups are very small (approximately 1 cm in total head and body length), and the inoculum was not introduced specifically (or uniformly) to a single location in the brain. It is possible that variable results were obtained because the virus was introduced into different regions of the brain among the inoculated animals. Future studies are planned to address these factors, analyze the organ distribution of ZIKV utilizing varying routes of infection (subcutaneous, intra-peritoneal, and intra-muscular), and determine viral load among organs in order to better characterize the model.

## Figures and Tables

**Table 1:** ZIKV replication following intra-cerebral inoculation of *M. domestica*.

Animals	Animal #1	Animal #2	Animal #3
Group 1 (1-day-old pups)	1.5 × 10^3^ PFU/g	n.d.	2.1 × 10^4^ PFU/g
Group 2 (2-day-old pups)	n.d.	n.d.	4.3 × 10^4^ PFU/g

Three pups in each of two litters (n = 6) of *M. domestica* were inoculated intra-cerebrally with 5000 PFU of ZIKV PRVABC59. At 20 days post-inoculation, animals in the two groups were euthanized, and the whole brain was collected, weighed, and homogenized for virus titration in Vero cells. Results are the means of duplicate samples. n.d. = no titer detected.

**Table 2:** Weights, anatomic measurements, and brain scores from ZIKV- and PBS-inoculated laboratory opossum pups.

Litter Number	Breeding Stock	ID Number	Sex	Inoculation Age (Days after Birth)	Equivalent Human Age (Wks. Post-conception)	Inoc. Vol. (µL)	Virus or PBS	Age at Harvest	Animal Weight (g)	Nose-Rump Length (mm)	Head Length (mm)	Head Width (mm)	Pathology
													NS1
1	PBP	O9378	F	4	8	2	V	78	48.3	117.7	35.6	17.6	3
													3
	PBP	O9379	F	4	8	2	V	78	48.6	116.2	34.2	17.6	0
													1
	PBP	O9380	M	4	8	2	V	78	59.1	123.0	36.9	18.5	1
													2
	PBP	O9381	M	4	8	2	V	78	56.8	117.4	35.9	18.4	3
													3
2	LL1	O9355	F	6	12	2	V	80	16.7	74.7	27.0	14.2	1
													2
	LL1	O9356	F	6	12	2	V	80	48.6	114.8	35.5	18.3	1
													3
	LL1	O9357	F	6	12	2	V	80	39.6	109.8	35.6	16.7	2
													3
	LL1	O9358	F	6	12	2	V	80	40.4	109.6	33.5	17.3	1
													3
	LL1	O9359	M	6	12	2	V	80	47.7	117.0	36.3	19.0	1
													2
3	FD2M	O9335	F	9	12.5	5	V	83	39.7	112.8	35.5	17.6	0
													1
	FD2M	O9336	M	9	12.5	5	V	83	38.3	106.6	34.5	17.1	2
													2
	FD2M	O9337	M	9	12.5	5	V	83	41.7	112.5	36.0	16.9	1
													3
4	LL1	O9248	F	20	20	10	V	94	52.4	124.3	38.0	18.4	1
													3
	LL1	O9249	M	20	20	10	V	94	72.8	134.9	40.4	19.2	1
													2
	LL1	O9250	M	20	20	10	V	94	55.2	123.3	39.4	17.9	1
													3
	LL1	O9251	M	20	20	10	V	94	66.0	132.0	40.1	19.2	3
													3
5	ATHHN	O9394	F	2	7	1	PBS	76	32.1	98.6	31.9	17.0	0
													0
	ATHHN	O9395	M	2	7	1	PBS	76	29.8	98.0	31.8	16.8	0
													0
	ATHHN	O9396	M	2	7	1	PBS	76	34.6	102.5	33.0	16.3	0
													0
	ATHHN	O9382	F	4	8	2	PBS	78	28.4	96.6	30.3	16.8	0
													0
	ATHHN	O9383	M	4	8	2	PBS	78	22.9	88.5	28.4	15.0	0
													0
	ATHHN	O9384	M	4	8	2	PBS	78	27.9	96.0	31.3	15.5	0
													0
	ATHHN	O9385	M	4	8	2	PBS	78	36.2	102.4	31.5	17.1	0
													0
6	LSD	O9339	F	9	12.5	5	PBS	83	32.8	99.4	29.6	16.8	0
													0
	LSD	O9340	F	9	12.5	5	PBS	83	34.4	99.9	32.8	15.8	0
													0
	LSD	O9341	M	9	12.5	5	PBS	83	33.6	100.4	31.2	16.9	0
													0

Note that weights and measurements among litters are not comparable because of different ages of harvest and differences among breeding stocks in growth rates. Brain pathology was scored subjectively on a scale of 0–3: 0, normal; 1, mild pathology; 2, moderate pathology; 3, extreme pathology (spongiform-like appearance). Brain NS1 levels (extent of fluorescent signal) were scored similarly: 0, none; 1, minimal; 2, moderate; 3, extreme.

## Data Availability

The data presented in this study are available on request from the corresponding author.

## Supplementary Materials

The following supporting information can be downloaded at: https://www.mdpi.com/article/10.3390/pathogens12050733/s1, Figure S1: In situ primer/probe sequences; Figure S2: Pathology/NS1 Scores.
