# Supplementary material for: Monodelphis domestica as a Fetal Intra-Cerebral Inoculation Model for Zika Virus Pathogenesis
Source: Pathogens. 2023 May 19;12(5):733. doi: 10.3390/pathogens12050733 (PMC10221844; doi:10.3390/pathogens12050733)
Supplement: Supplementary file 1 [file pathogens-12-00733-s001.zip › pathogens-2350194-supplementary.pdf]

pathogens

MDPI

Supplemental Figure S1. In situ primer/probe sequences.

Zika NS5 Protein Forward: (CGCATTGAAAGGATCCGCAG)

Zika NS5 Protein Reverse: (TCCTTCCTCCTGGTATGCGA)

NS5 Probe Sequence:
CGCATTGAAAGGATCCGCAGTGAGCACGCGGAAACGTGGTTCTTTGACGAGAACCACCCATAT
AGGACATGGGCTTACCATGGAAGCTATGAGGCCCCACACAAGGGTCAGCGTCCTCTCTAATA
AACGGGGTTGTCAGGCTCCTGTCAAAACCCTGGGATGTGGTGACTGGAGTCACAGGAATAGCC
ATGACCGACACCACACCGTATGGTCAGCAAAGAGTTTTCAAGGAAAAAGTGGACACTAGGGTG
CCAGACCCCCAAGAAGGTACTCGTCAGGTTATGAGCATGGTCTCTTCCTGGTTGTGGAAAGAG
CTAGGCAAACACAAACGGCCACGAGTCTGTACCAAAGAAGAGTTCATCAACAAGGTTCGTAGC
AATGCAGCATTAGGGGCAATATTTGAAGAGGAAAAAGAGTGGAAGACTGCAGTGGAAGCTGT
GAACGATCCAAGGTTCTGGGCTCTAGTGGACAAGGAAAGAGAGCACCACCTGAGAGGAGAGT
GCCAGAGTTGTGTGTACAACATGATGGGAAAAAGAGAAAAGAAACAAGGGGAATTTGGAAAG
GCCAAGGGCAGCCGCGCCATCTGGTATATGTGGCTAGGGGCTAGATTTCTAGAGTTCGAAGCC
CTTGGATTCTTGAACGAGGATCACTGGATGGGGAGAGAGAACTCAGGAGGTGGTGTTGAAGGG
CTGGGATTACAAAGACTCGGATATGTCCTAGAAGAGATGAGTCGCATACCAGGAGGAAGGA

Supplemental Figure S2

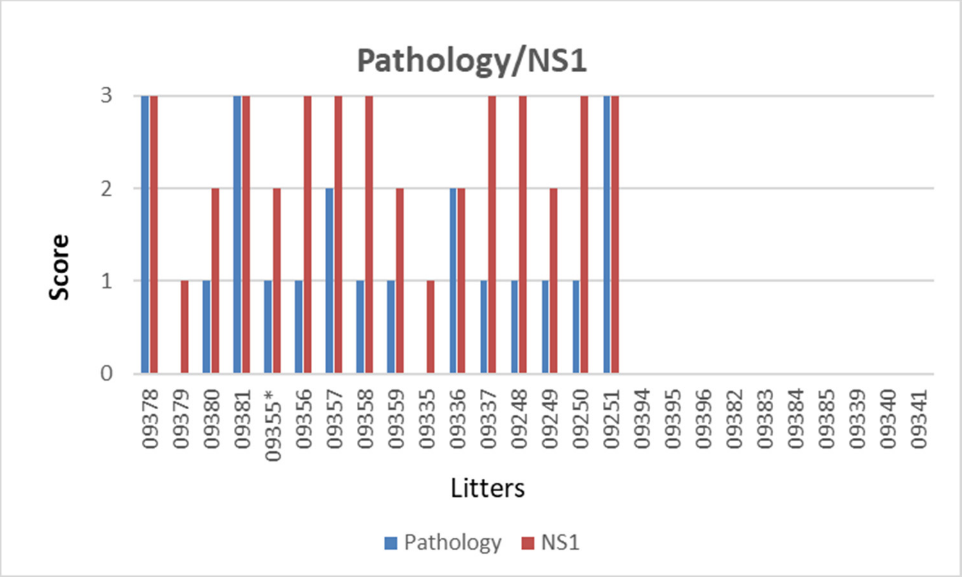

Supplemental Figure S2. Pathology and NS1 brain scores from ZIKV-inoculated and PBS-inoculated laboratory opossum pups. Blue bars represent pathology (scored from 0-3), and red bars indicate NS1 expression (scored from 0-3). The absence of a bar indicates a score of 0. The ten pups indicated to the right-hand side were injected with PBS. The * denotes the growth-restricted pup, O9355.
